# Risk factors for rehospitalization and inpatient care among pediatric psychiatric intake response center patients

**DOI:** 10.1186/1753-2000-8-27

**Published:** 2014-10-29

**Authors:** Krystel Tossone, Eric Jefferis, Madhav P Bhatta, Sumru Bilge-Johnson, Patricia Seifert

**Affiliations:** Kent State University, Lowry Hall, 750 Hilltop Drive, PO Box 5190, Kent, OH 44242 USA; Kent State University, 319 Lowry Hall, PO Box 5190, Kent, OH 44242 USA; Akron Children’s Hospital, 388 S. Main St. Ste. 207, Akron, OH 44311 USA; Akron Children’s Hospital, Akron, OH 44311 USA

**Keywords:** Pediatric, Hospital psychiatric departments, Risk factors, Inpatient, Relapse

## Abstract

**Background:**

The study sought to explore the characteristics, risk factors for inpatient recommendation, and risk factors for revisits to a pediatric psychiatric intake response center (PIRC). There are three research questions: 1. What is the general profile of pediatric patients who present at the PIRC? 2. What are the risk factors for patients who repeatedly visit the PIRC? 3. What are the risk factors for PIRC patients who are recommended to inpatient care?

**Methods:**

The study utilized a retrospective medical chart review of a random sample (n = 260). A PIRC profile was created using frequency and prevalence calculations, in addition to a survival analysis of patients who return to the PIRC in order to determine how long it takes for PIRC patients to return to the PIRC. Factors that contribute to increased odds of returning to PIRC and being recommended for inpatient treatment were calculated using two logistic regression analyses.

**Results:**

The average pediatric PIRC patient is about 13 years old, Caucasian, with Medicaid and comes from a divorced or single parent household. About 43% of patients presented at PIRC for suicidal thoughts, ideation, intentions or actions. At least 63% of patients have a history of victimization. The average time to return to PIRC is about 90 days. Patients with a history of victimization, suicidal behavior, learning problems, problems with peers, and a history of violence were at an increased odds of returning to the PIRC. Those patients who were previously admitted to inpatient care and had a family history of mental health issues were at increased odds of being recommended to inpatient treatment.

**Conclusions:**

This sample presents with a multitude of issues that contribute to increased odds of revisits to PIRC and inpatient recommendation. These issues seem to come from multiple levels of influence. Future research should expand to similar treatment facilities and use a prospective design to confirm risk factors. Treatment for pediatric psychiatric patients may focus on multiple factors that influence patients’ mental health.

## Background

Youth mental health illness is a widespread health problem in the United States. Over 46% of adolescents report a mental health disorder in their lifetime and 21% of those adolescents report a serious mental health disorder [1]. Many of the determinants of pediatric psychiatric disorders are exogenous. For example, family and community risk factors, such as parental mental health disorders, neighborhood stress, family satisfaction, and school stress increase the odds of a child having a psychiatric disorder [2]. The effects of pediatric mental health issues and adverse childhood experiences extend into adulthood. The Adverse Childhood Experiences Study examined the multiple childhood risk factors for adulthood problems, finding that adverse childhood experiences such as abuse, family mental health issues, or witnessing violence were strongly associated with poor physical health and mental health disorders in adults [3]. A longitudinal study examined a more direct relationship between childhood psychotic symptom reporting and adult schizophrenia, finding that reporting psychotic symptoms as a child contributed 42 percent to the risk of reporting schizophreniform symptoms as an adult [4]. Suicide is a devastating outcome of mental health disorders among youth. The Centers for Disease Control and Prevention (CDC) [5] reports a rate of 4.5 suicides per 100,000 persons aged 10 to 19 years, making it the third leading cause of death for children and youth.

Psychiatric issues among children and adolescents cause considerable burden not only to patients and their families but also to the healthcare system. Emergency Departments (ED) treat about 326 pediatric patients per 100,000 for psychiatric issues annually [6]. Psychiatric pediatric visits in the ED take about 5 hours on average and are taxing on emergency departments’ resources [7, 8]. ED providers report shortcomings with EDs in regards to juvenile psychiatric visits including inadequate psychiatric evaluations and long waits for evaluation and placement [8]. Another undesirable outcome of ED visits for children with mental health complaints is inpatient care. Inpatient care is incredibly costly to the hospital. The average annual cost per inpatient is $5,385 compared to $937 for annual outpatient care [9]. Inpatient care, while sometimes appropriate for the pediatric patient, is costly and should only be used for the most serious psychiatric disorders, such as suicidal behavior or psychotic disorders [10, 11].

Regardless of the treatment, it should be the best fit for the patient and determined by providers who are trained in psychiatric evaluation [7]. General EDs may not be the best places to present for mental health conditions. Christodulu et al. report that patients attend EDs mostly because it was their only option and community resources were unavailable or unknown [8]. If EDs are the only option in some communities, they should be equipped to treat pediatric psychiatric patients. It is recommended that a mental health diagnosis tool, such as the Children’s Global Assessment Functioning scale [12], be employed in medical settings. However, they are not employed as widely as they should be in medical settings [10]. Other screening tools have been developed for children, for example, Horowitz et al. developed a reliable suicide screening tool that can be used in EDs [13]. Advanced screening tools such as this one may help providers to triage care to the appropriate department.

Another avenue to address reduction of ED visitation time for mental health issues, and triage the right mental health complaint to the right mental health treatment outcome, may be psychiatric response centers within the pediatric emergency department. Triaging care in the EDs is a classic aspect in emergency medicine, partly to address the growing numbers of patients who attend EDs versus the limited resources of hospitals and partly to direct appropriate care [14]. The Psychiatric Intake Response Center (PIRC) examined in this study triages care for youth who present at the ED or make an appointment, resulting in three outcomes: Inpatient admission (the hospital’s inpatient ward or another psychiatric center), outpatient treatment, or partial hospitalization. The PIRC also provides linkage to services in the community and specialized programs. Recognition of a lack of post-discharge services as a risk factor for pediatric psychiatric repeated admission [15, 16] and the need for an isolated triage prompted the creation of a children’s hospital psychiatric intake response center.

One of the most restrictive treatment modalities of mental health disorders is inpatient admission among youth, an outcome PIRC seeks to reduce. Readmission is a significant problem in pediatric psychiatric inpatient admission. The average youth psychiatric inpatient readmission rate ranges from 30% to 60% [15, 17, 18]. At least one-third of children and adolescents are more likely to be rehospitalized within the first 3 months to two years after the first admission [15–17]. Those who are at a younger age at admission are generally more likely to be readmitted [17, 19, 20]. Medical nonadherence and polypharmacology is associated with readmission [19, 21, 22]. Post-discharge services decrease risk of readmission and lack of services increases risk of readmission [15, 23]. Structural and geographic factors also play a significant part in increasing risk of psychiatric rehospitalization. Those living in foster care and congregate care are also more likely to be rehospitalized [24], and those who live in rural areas are more likely to be rehospitalized [23].

The PIRC also aims to reduce returners (>1 visit annually) to its facility. Combined with rising ED visit rates, returning patients to the ED represent a growing problem. A statewide study found that repeat patients compose about two-thirds of ED patients [25]. A study of Belgian psychiatric adult patients examined time to revisit the ED [26] and reported that the majority of patients returned within a few weeks, and younger patients (0 to 44 years) were more likely to return sooner than older patients (45 years and older). About 21% pediatric psychiatric ED patients are repeat visitors [27]. Also, repeat visitors were more likely to present for suicidal issues and have prior hospitalizations than those who visited the ED once. These results in combination with the results of another study, where 40% of pediatric psychiatric emergency service visits were classified as non-urgent [28], may point to a need to efficiently address pediatric psychiatric care in the ED. Triage centers for pediatric psychiatric care may be the answer.

To date, these triage centers have received very little scientific attention and little is known about the population being served, treatment of the population, and related risk factors. A comparable service seems to be a pediatric emergency service (PES), yet this service is also understudied [28]. This article addresses the literature gap regarding pediatric psychiatric response centers and comparable services by presenting a population profile and investigating risk factors for revisits to the Psychiatric Intake Response Center (PIRC) and readmission into inpatient care using retrospective chart review. The study sought to answer three research questions: 1. What is the general profile of pediatric patients who present at the PIRC? 2. What are the risk factors for patients who repeatedly visit the PIRC? 3. What are the risk factors for PIRC patients who are recommended to inpatient care? The following sections discuss the methods used to answer the research questions, analytic results, and implications of research.

## Methods

### Sample

The site is housed within a Midwest pediatric hospital in an urban area close to multiple diverse neighborhoods. The PIRC treats about 1,200 children ages 0 to 19 years annually. It serves pediatric patients in the immediate surrounding area and patients in suburban and rural neighborhoods. It is composed of a separate triage unit within the ED that uses a quick risk assessment system to determine outcome recommendations for pediatric patients presenting with psychological and behavioral issues. Subjects may attend PIRC from the ED (ambulance, police or caretaker-accompanied), referral from another physician, or by appointment. PIRC patients with psychological or behavioral complaints are in a separate area from general ED patients, with the goal of maintaining more confidentiality and a calmer environment for patients and their families. A case worker conducts an interview with the patient and/or his or her caretaker to assess the patient’s risk of harm to self or others.

The sample included patients who presented at PIRC from 2010 through 2011 (n = 261). The scope of the study used a two-year time frame to investigate the prevalence of returning patients to the PIRC. Studies of pediatric patients presenting to emergency rooms and inpatient hospital admissions found periods from 30 days to 5 years for returning patients [15, 17, 18, 21], therefore a two-year period seemed a reasonable middle ground for investigation. The study employed a random systematic ten percent sampling frame of paper medical charts. Inclusion criteria required patients to be 18 years of age or younger and visited the PIRC at least once in 2010–2011. Institutional Review Boards of the Kent State University and Akron Children’s Hospital reviewed and approved the study protocol in May 2012. A HIPAA waiver was received in the Institutional Review Board application due to the nature of the study (a medical chart review without identifiers).

### Instrument

The 10-page risk assessment contains categories to assess background of the patient, treatment and psychological history, and risky behaviors. The risk assessment is conducted by a case worker and approved by the psychiatrist-on-call. For many of the items on the risk assessment there is not a scale that determines whether the patient is placed in a category or diagnosed, with the exception of the suicide risk assessment tool that tallies up a score to determine whether the patient is a risk to him or herself or others. The lack of psychometric scales for determination of issues such as victimization or depression is due to the PIRC’s philosophy to seek the quickest way to triage psychiatric care and less on fully diagnosing and profiling patients while they are at the triage center. The assessment queries current and past mental health treatment including medication history, abuse history, substance use history, sexual history, impulsive behavioral history (such as running away, suicidal behavior, self-harm behavior such as cutting, violent behavior, fire setting, and animal abuse), other factors that lead to a global assessment functioning score, and final outcome recommendation (inpatient, outpatient or partial hospitalization). This study was most interested in risk factors for inpatient recommendation, and in our case inpatient recommendation by the case worker and psychiatrist-on-call resulted in 95 out of 98 patients (97%) complying with the recommendation for admission either into the hospital’s own inpatient care or another local inpatient hospital.

The retrospective chart review in a psychiatric setting followed the format outlined by Gearing and others [29]. The first part of the study involved conceptualizing the aims and hypotheses; a primary aim of the study was developing a comprehensive profile of patients who attend the PIRC. This part of the study also included a literature review, variable selection and operationalization following examination of five randomly selected charts. Examination of initial charts also determined the organization of data by mimicking the chart’s style in a Microsoft Access Database.

An important step focused on creating rules and protocol for data entry and organization. There was one data abstractor in this study. All data for that patient would be collected for multiple dates. Multiple imputations were reconciled by creating historical variables. For example, one patient may have multiple conflicting imputations of “sexual abuse”: One date may indicate “yes” and another may indicate “no”. The rule stated that in the case of multiple conflicting imputations over the course of a patient’s chart history for a variable, the true value would be “yes” if any of those records stated “yes”. This created a historical variable, that is “history of sexual abuse” for that patient because at some point in the patient’s medical record, one of the charts indicated that he or she had a sexual abuse experience, even if previously or after that experience the case worker may not have indicated that experience happened (either because it was prior to the experience happening or because the case worker could not get the respondent to respond or be truthful about his or her experiences). We applied this method to the social behavioral variables, the outpatient treatment variable, and known diagnosis variable. We did not employ this method to the outcome recommendation variable. In this case, we used the earliest record for a patient’s chart as the indicator for outpatient, inpatient or partial hospitalization. We did not use the last record as we were concerned about potential bias toward those returners who had an inpatient recommendation after several outpatient recommendations versus a patient with only one recommendation that was an inpatient recommendation.

Most of the items on the assessment, with the exception of the demographic variables and Global Assessment Functioning Score variable, were binary (yes or no) with follow-up open-ended responses. The first part of the assessment queries about patient-reported current and past psychological treatment. This concerns “history of inpatient outcome”- patient reports having a previous admission to an inpatient unit at a hospital, “known past diagnosis”- patient reports a previous mental health diagnosis such as depression, and “current treatment”- patient reports receiving current outpatient mental health treatment, “reason for referral”- patient reports why he or she is at the PIRC, and “Family History of Mental Health Issues”- patient reports whether there is a history of mental health problems, who has those issues, and what those issues are. The next part of the assessment queries about victimization and legal problems. The case worker queries whether the patient has experienced any of the following victimizations: Sexual abuse, physical abuse, emotional abuse, neglect, witness to violence, and other trauma. These binary questions also have follow-up open-ended responses in order to examine, for example, what type of witness to violence is present. Bully victimization is determined as a yes or no question in a later section, the education section of the assessment. This section of the assessment asks whether a patient has “peer problems”, “motivation problems”, “homework problems”, and “attention problems”. Another important part of the assessment queries about behavioral problems such as self-harm, suicidal, and violent behaviors. The case-worker and psychiatrist on call differentiates “self-harm” from “suicidal” behavior by whether or not the patient intends to kill him or herself, thinks about killing him or herself, or plans to kill him or herself. For example, a patient may cut him or herself, however if he or she is not doing this with thoughts, plans or intentions for suicide, this is determined to be “self-harm” behavior. It should be noted that some of our sample had both a positive history for self-harm and a positive history for suicidal behavior. Again, most of the assessment relies on patient self-report or parent report.

### Analysis

Statistical analysis employed descriptive calculations including frequency and proportions, means, and standard deviations in developing a general profile of PIRC patients. Kaplan-Meier survival analysis was employed to estimate time to revisit the PIRC. In preparation of the survival analysis, a variable was created using the dates of the visits to PIRC to estimate the number of days from first visit to second visit, second visit to third visit, and so on. The “time in days” variables allowed for a probability estimate of how long it would take a patient to come back to the PIRC after the first time, second time, and so forth. In the final survival analysis, patients who did not return to the PIRC (n = 211) were excluded from the study because the focus was on how long it would take returners to come back to the PIRC, not on the general PIRC population (n = 49). While a general survival analysis employs the entire study population to determine how many “fail” (or in our case come back to the PIRC), our study was more focused on when those failures occurred. The concern was if we include all of the population, it would skew the average time to return by including those that were censored as part of the mean calculation. In fact, when comparing both the full sample survival analysis and the sub-sample survival analysis, having 81.6 percent of the study as “censored” resulted in skewing the model toward an average survival time of 420 days.

Chi-square analyses and a logistic regression were conducted to examine factors that increased the odds of returning to the PIRC. Chi-square analyses were first conducted for each hypothesized exposure variable in relationship to whether a patient only visited the PIRC once or if the patient visited more than once in the 2010–2011 time frame. Hypothesized exposure variables were included based on previous literature of revisits to emergency departments and interest: History of depression, history of inpatient outcome, suicidal behavior, self-harm behavior, violence history, peer problems, learning problems, victimization, and family history of mental health issues. Significant variables were included in the logistic regression model and multicollinearity tests of statistical moderation were conducted. The regression model is presented as unadjusted and adjusted for age, gender and race.

A similar process was used to determine which variables increased odds of being admitted to inpatient care. First, Chi-square analyses were conducted for each hypothesized exposure variable in relationship to admission recommendation. Hypothesized exposure variables were included based on previous literature and interest: Previous admission, family history of mental health issues, victimization history, revisits to PIRC; presence of past diagnosis, suicidal behavior, self-harm behavior, violent behavior and history of depression. If there was a significant association, those variables were included in the logistic regression model to address whether those variables were significant predictors for outcome recommendation (inpatient or outpatient). Because of the small sample size of partial hospitalization outcome patients combined with a lack of statistically significant association with all exposure variables during chi-square analyses, that outcome was excluded from the analysis. Moderation effects were examined using multicollinearity tests for interaction. All analyses were conducted using Statistical Analysis Software (SAS) version 9.2.

## Results

Table 1 shows demographic, psychiatric and social behavioral variables for the sample. The average patient presenting at the PIRC is about 13 years old, Caucasian with Medicaid and comes from a divorced or single parent household. About 21 percent of patients have been to the PIRC at least twice in a two-year period, and most are recommended for either outpatient or inpatient treatment. Most of the patients (about 43%) present at PIRC for suicidal thoughts, ideation, plan or attempt. Nearly half of patients have a history of at least one kind of harmful behavior and nearly 75% of patients experienced some form of victimization with 18% of patients experiencing more than one kind of victimization.

**Figure 1:**
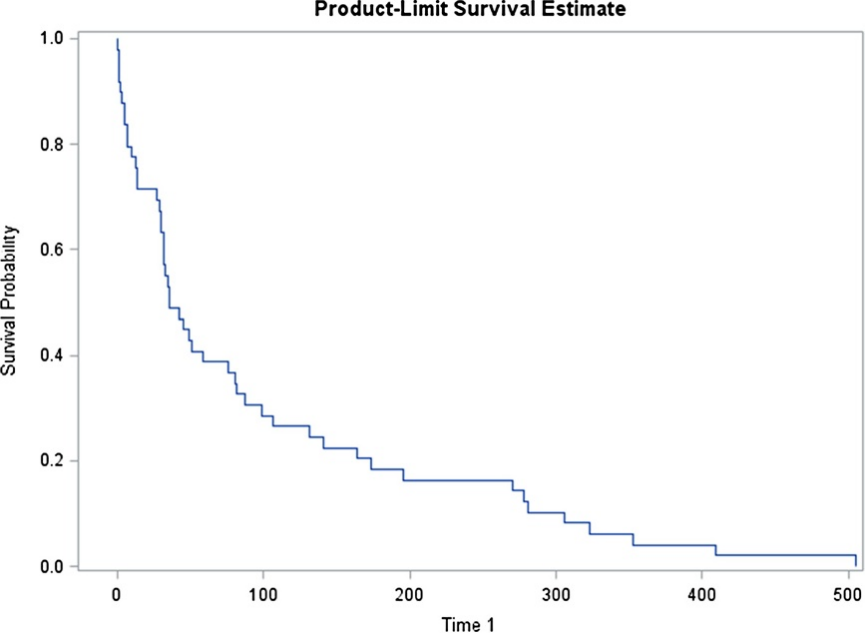
**Survival estimate of time in days to return to the psychiatric intake response center; 50 percent return by day 36 (n = 49).**

Because of the relatively high percentage of revisits and interest in the revisit population, Kaplan-Meier Survival Analysis was employed to determine the average time for revisiting the PIRC among returners (n = 49). Figure 1 shows the Kaplan Meier Survival Analysis of time in days to return to the psychiatric intake response center among returners. The mean time to return to the PIRC, for those who return, was about 96 days (std. dev. 17.49 days). Examination of the median quartile estimates finds that by 14 days, 25% of the returners have returned, by 36 days 50 percent of the returners have returned and by 131 days, 75 percent of the returners have returned.

**Table 1:** **Demographic**, **psychiatric and social behavioral characteristics of psychiatric intake response center patients at mid**-**western children**’**s hospital**, **2010**-**2011** ^**1**^

Characteristics	Percent (Frequency)	Mean
***Demographic***		
**Gender** **(n** = **260)**		
Male	50.38% (131)	
Female	49.62% (129)	
**Age** **(n** = **260)**		13.43
<= 12 Years	32.3% (84)	
13-14 Years	22.3% (58)	
15-16 Years	26.1% (68)	
> = 17 Years	19.2% (50)	
**Race/Ethnicity** **(n** = **256)**		
Caucasian	79.69% (204)	
African-American	14.45% (37)	
Other^2^	6.86% (15)	
**Insurance status** **(n** = **240)**		
Medicaid	53.75% (129)	
Private	32.08% (77)	
More than one type	10.00% (24)	
Self-Pay	4.17% (10)	
**Family structure** **(n** = **250)**		
Divorced/Single parent	58% (145)	
Two parent household	30.8% (77)	
Foster, group home, jail	6% (15)	
Family guardian	5.2% (13)	
***Psychiatric***		
**No. of visits to PIRC** **(n** = **260)**		1.36
1 Visit	79.62% (207)	
2-10 Visits	21.38% (53)	
**Current treatment** **(n** = **260)**		
Yes	45.38% (118)	
No	54.62% (142)	
**Known past diagnosis** **(n** = **260)**		
Yes	68.85% (179)	
No	31.15% (81)	
**Reason for referral** **(n** = **260)**		
Suicidal	42.91% (109)	
Behavioral issues^3^	18.50% (47)	
Mood-Related issues^4^	18.50% (47)	
Aggression/Homicidal	7.87% (20)	
Self-Injury	6.69% (17)	
Substance use^5^	3.15% (8)	
Psychotic/Delusional	2.36% (6)	
**Outcome recommendation** **(n** = **260)**		
Outpatient	41.15% (107)	
Inpatient	37.69% (98)	
Partial	20.38% (53)	
**Global assessment functioning score (n** = **260)**		33.63
10-20	32.18% (84)	
21-30	16.86% (44)	
31-40	31.42% (82)	
41-50	14.56% (38)	
51-65	4.98% (13)	
***Social behavioral***		
**Harmful behaviors** **(n** = **260)**		
Suicidal	53.08% (138)	
Self-Harm	48.46% (126)	
Violence or destruction	48.46% (126)	
Homicidal	9.23% (24)	
Animal abuse	9.62% (25)	
**Substance use** **(n** = **259)**		
Yes	22.73% (59)	
No	77.22% (200)	
**Victimization** (**n** = **260**)		
None	37.69% (98)	
Bullied	24.62% (64)	
Physical abuse	20.38% (53)	
Sexual abuse	18.08% (47)	
Emotional abuse	11.54% (30)	
Neglect	9.23% (24)	
Witness to violence	21.15% (55)	
Multiple types	18.22% (47)	
**Educational** **(n** = **260)**		
Concentration problems	43.08% (112)	
Attention problems	40.00% (104)	
Ind. education plan	32.31% (84)	
Motivation problems	27.69% (72)	

^1^Not all items add up to 100% due to categories not being mutually exclusive for some individuals.

^2^”Other” includes Hispanic/Latino, Asian, Native American, and unspecified other.

^3^“Behavioral Issues” includes “Behavioral”, “Out Of Control (OOC)”, “Run away”, “Eating disorder”, “Psychiatric care”.

^4^“Mood-Related Issues” includes “Anger”, “Mood swings”, “Depression”, “Anxiety”.

^5^“Substance Use” includes “drug use”, “ingestor”, “meds”, “OD/Overdose”, “Withdrawal”.

About 21% of patients returned to the PIRC within a year. Table 2 shows the result of the logistic regression modeling the odds that a patient will return to the intake center within a year of first arrival. There were many variables included in this model. No interaction effect was found between any of the exposure variables. Having suicidal behavior, victimization history, peer problems, history of violence, and learning problems were found to increase the odds of returning to the intake center, in some cases 5 times the odds. Even though the model was adjusted, no confounding effect was found between the demographic variables and the exposure/outcome variables. However, some of the odds did increase for a few of the variables with the introduction of the demographic variables, particularly age.

**Table 2:** **Logistic regression model predicting pediatric returns to the psychiatric intake response center**

	Odds ratio	95% CI	Df	P-value	R ^2^
***Unadjusted model***			4		0.18
Suicidal behavior	**3.734**	**1.646**-**8.473**		**0.001**	
Victimization history	**2.882**	**1.346**-**6.174**		**0.006**	
Peer problems	**2.875**	**1.411**-**5.859**		**0.003**	
History of violence	**2.161**	**1.040**-**4.488**		**0.038**	
Learning problems	**4.668**	**1.338**-**16.283**		**0.015**	
***Adjusted model***			8		0.19
Suicidal behavior	**4.218**	**1.784**-**9.973**		**0.001**	
Victimization history	**2.782**	**1.285**-**6.020**		**0.009**	
Peer problems	**2.671**	**1.278**-**5.583**		**0.009**	
History of violence	**2.482**	**1.134**-**5.433**		**0.023**	
Learning problems	**5.254**	**1.485**-**18.585**		**0.010**	

Note: **Bold** font numbers indicate statistical significance at p ≤ .05.

In regards to outcome recommendation, 41.15% of patients were recommended for outpatient treatment, 37.69% were recommended for inpatient admission, and 20.38% were recommended for partial hospitalization. Table 3 shows the result of the logistic regression modeling the odds that a patient is recommended for inpatient services. Adjusting for age, gender and race/ethnicity variables, those who had a history of inpatient admission and family history of mental health issues were at significantly greater odds (2.143 and 3.370, respectively) for inpatient recommendation. An interaction effect was found between suicidal behavior and self-harm behavior, meaning that patients who self-harm and exhibit suicidal behavior are at magnified odds for being recommended for inpatient treatment than patients who exhibit one of the behaviors alone. It should be noted that demographic variables were not found to be significant contributors to inpatient recommendation, but were considered for the adjusted model because they may have had an effect on the exposure variables. However, this was not found to be the case for this model.

**Table 3:** **Logistic regression model predicting inpatient outcome recommendation at the psychiatric intake response center**

	Odds ratio	95% CI	Df	P-value	R ^2^
***Unadjusted model***			5		0.129
Previous admission	**2.209**	**1.185-** **4.118**		**0.012**	
Family history of mental health problems	**3.300**	**1.445-** **7.538**		**0.004**	
History of victimization	1.328	0.764-2.308		0.314	
Suicide*Self-Harm	1.356	0.545-3.379		0.069	
***Adjusted model***			8		0.14
Previous admission	**2.143**	**1.137-** **4.040**		**0.018**	
Family history of mental health problems	**3.370**	**1.459-** **7.785**		**0.004**	
History of victimization	1.240	0.701-2.195		0.311	
Suicide*Self-Harm	0.460	0.210-1.009		0.061	

Note: **Bold** font numbers indicate statistical significance at p ≤ .05.

*Indicates a multiplicative interaction term.

## Discussion

The PIRC patient population is similar to samples of pediatric psychiatric patients presenting at emergency departments; Sills and Bland [6] report that the majority of their sample of emergency department patients are 13 to 18 years of age and the gender of patients is nearly equal.

Consistent with Santiago et al., the majority of pediatric patients in our sample are seen for suicidal intention, thoughts, plans or actions. Santiago et al. [7] reported suicidal ideation as the reason for referral in 39% of their emergency department pediatric sample [7]. In a study of adult PES users, about 55% exhibited suicidal ideation, similar to our population [30]. Part of the PIRC risk assessment process requires that if a patient presents with suicidal or homicidal thoughts or intents and cannot sign a safety agreement contract, then the patient is recommended to go into inpatient treatment. In a 2009 study, two-thirds of adolescents and children who committed suicide had no history of seeking mental health services a month prior to their suicide, a statistical difference compared to children who did not commit suicide [31]. While a large number of the patients in our study presented for suicidal behaviors, it may be that pediatric patients who are presenting as suicidal feel as though they can use the PIRC as a mental health support system.

Risk factors for revisits to PIRC were numerous. Suicidal behavior, victimization history, peer problems, history of violence, and learning problems were significant risk factors, even after adjustment for possible covariates or confounders. Further exploration of the possible risk factors for revisits is needed. It may make sense to focus on the first risk incurred 90 days after initial visits, as it seems that this is when most revisits occur. Because this is a sample with little history in the literature, it is difficult to compare to other studies regarding children revisiting for psychiatric issues. A study of a PES examined urgent emergencies among children, but did not gather data on revisits [28]. Another similar sample may be pediatric patients who present at the emergency room multiple times for a psychiatric complaint. Peterson et al.’s examination (1996) of risk factors for pediatric psychiatric emergencies discovered that younger age at initial visit and presentation during the school year significantly predicted multiple ER visits [11]. While our study did not find age to be a significant predictor on its own, age may be related to some of the predictor variables such as victimization.

Two interesting significant exposures that increased the odds of revisits were presence of victimization history and occurrence of learning problems. Children with victimization history were nearly 4 times as likely as children without a victimization history of returning to the PIRC within the two-year span. It is well-known in the literature that in the general pediatric population, children who are victimized often develop mental health issues during childhood and into adulthood [3, 32]. Children with learning problems such as attention problems, motivation problems, being on a learning disability learning plan (such as a 504 plan), had nearly 5 times the odds as children who did not report any of these problems to revisit the PIRC. This may be related to peer victimization such as bullying in schools. Other studies have explored this relationship [33, 34].

Previous admission and family history of mental health issues are significant risk factors for inpatient recommendation in this sample. Previous admission is an important predictor of subsequent inpatient admission in other studies of emergency care [17, 18, 35]. A study conducted in a general PES for factors affecting inpatient hospitalization found different risk factors for inpatient admission than our sample exhibited: Clinical severity, age, gender, race, homelessness, and employment status were significant predictors [30]. However, this was an adult population in a different geographic setting than our sample and may not be comparable. Furthermore, relapse in patient admission is not uncommon. Fontanella (2009) report that 38% of inpatient subjects relapsed within a year, and most within 3 months after discharge [22]. Children with severe psychotic episodes have been shown to fare worse: About 60% have relapsed [21]. Our study did not examine risk factors for inpatient rehospitalization in this sample due to a small size of the subset. Further research using a larger sample size aims to focus on the recurrent inpatient population.

Another risk factor for inpatient recommendation in this sample is family history of mental health issues. Subjects who reported family history of mental health issues had at least 3 times greater odds of inpatient recommendation than those who did not (P = .0004). This is not a surprising result given the outcomes of the Adverse Childhood Experiences Study (ACES). A segment of the study found exposure to family mental health disorders as a child increases risk of multiple negative health outcomes as an adult, pointing to the conclusion that adverse childhood mental health exposure is long lasting [3]. In addition to ACES, other studies examined caregiver issues as an increased risk factor for inpatient admission and visits to the emergency department for children [16, 36]. It may be that mental health factors of family members, particularly caregivers, might affect the outcome recommendation for a number of reasons: Issues at home may result in providers wanting to give patients a ‘break’ from home or children who have parents with mental health issues may not be getting the care they need to handle their own issues let alone their children’s issues.

## Conclusions

This study focused on creating a profile of patients who attend the PIRC and examining revisits to the PIRC and recommendations to inpatient care. These two outcomes are the extreme situations that psychiatric emergency care may seek to decrease in the pediatric population. We sought to find risk factors for these two outcomes, and found them to be in multiple areas of practice. Practitioners in the field may want to focus on multiple risk factors for inpatient recommendation and revisit reduction that may incur within two to three months after the first visit.

There are limitations to this study. The first limitation involves using chart review. The charts were hand-written and occasionally illegible, and the case workers who fill out the chart reviews are assessing their patients’ risk, not gathering systematic review data. However, as Gearing et al. [29] note, more psychiatric research utilizing retrospective chart review as a methodology needs to be conducted, and it is a good first step in guiding clinical research because it is inexpensive and the data are easily available and in great quantities, allowing for hypothesis generation and the studying of rare occurrences. Also, the study should have employed two data abstractors, rather than one, to confirm the methodology of the data collection process. Because this was an exploratory study with limited funding, only one abstractor was viable, however, future studies focusing on chart review should employ at least two abstractors to reconcile the data. Another limitation relates to sample size and generalizability to other studies. Because this was a pilot study, the sample size was relatively small and may not be nationally representative of other psychiatric emergency pediatric patients. The sample size was too small for some variables to be explored; for example, children who live in group homes. Further research should focus on gathering a larger sample size to examine some of these possible risk factors.

The study contributes to the body of knowledge in a number of ways; first being that it fills a gap in the literature in regards to the sample population. While studies have discussed screening for mental health issues in the emergency department [13, 37, 38], there are few studies that focus on this type of pediatric psychiatric service. Compared to similar studies of repeat pediatric psychiatric visits to the ED, we found a similar return prevalence of about 30%. Other studies found a higher return prevalence, perhaps because the setting was a general ED and not a specialized mental health treatment ED, perhaps indicating that a PIRC system decreases return psychiatric ED visits in the pediatric population. Additional research will need to explore this possibility. A similar population may be the PES population. The PIRC utilizes a combination of suicide screening, on-call therapist, specialized staff, in addition to other services similar to those services employed by a PES. This study may serve to inform similar intake centers as to what risk factors may be a target for research to decrease inpatient recommendation or revisits. Similar to our study, Goldstein et al. in 2007 found that those with prior psychiatric hospitalization and suicidal issues experienced increased return visits to the ED [27]. Focusing on those with prior inpatient hospitalization and suicidal issues in a PIRC setting may reduce repeat visits. Also, multiple exposure variables were collected, which allowed us to begin examining the larger relationships that exist among multiple variables. As shown, there are multiple variables that influence inpatient outcome recommendation and revisits in this population. Understanding how they work together may be the next step in the analysis.

Response and triage centers such as the one being discussed may be a less burdensome route for families to take when bringing children to the hospital for psychiatric issues than the emergency department. To the best of the authors’ knowledge, there are few studies that discuss a triage center for pediatric patients presenting for mental health issues. More work needs to be conducted to evaluate the center’s contribution to increasing the quality of care for psychiatric pediatric patients and how to reduce inpatient referrals and reduce revisits to the triage center. Further research should focus on the availability of similar centers across the United States in order to start compiling and evaluating best practices for these types of centers.

Due to the nature of this relatively exploratory study, there are many future avenues for pediatric mental health inpatient and revisit reduction research. We propose that further research focus on a few areas: The first would be expansion of the pilot study to include several years of data following patients with varying recidivism rates, in order to determine the full profile of risk factors for revisits and inpatient recommendation. Another avenue would be evaluating the intake center’s contribution to reduction of inpatient care and negative outcomes through retrospective case analysis. Also, cross site comparisons to determine if the PIRC model improves outcomes compared to more traditional PES would be valuable. Finally, research may expand beyond chart reviews and evaluation and use prospective methods.

Finally, there are recommendations for application of our results in a psychiatric emergency service or triage. Therapeutic and community programming may focus on a more systemic approach: Consideration of family mental health issues, victimization at home, in the community and schools, and learning problems may lead to decrease in hospital recidivism and inpatient care. Also, timeliness in follow-up and linkage to services for pediatric patients may be important given that 50 percent of returners do so by day 36 and the mean time to return is 96 days. Finally, due to the considerable financial burden on the health care system, as well as families, continued research should focus on further elaboration on community and family-based risk factors. Taking an ecological approach to treatment by considering factors such as individual (e.g. genetic), interpersonal (e.g. relationship with parents), structural (e.g. exposure to community or school violence), organizational (e.g. policies and procedures), and social factors (e.g. social norms, broader community issues), may create multiple avenues to address this problem and require medical providers to work with community members.
